# Trends in Tuberculosis Incidence in the Australian-Born in Victoria: Opportunities and Challenges to Elimination

**DOI:** 10.3390/tropicalmed3040112

**Published:** 2018-10-11

**Authors:** Ouli Xie, Ee Laine Tay, Justin Denholm

**Affiliations:** 1 Victorian Infectious Diseases Service, Royal Melbourne Hospital, Parkville, VIC 3050, Australia; ouli.xie@mh.org.au; 2 Department of Health and Human Services, Melbourne, VIC 3000, Australia; eelaine.tay@dhhs.vic.gov.au; 3 Victorian Tuberculosis Program, Melbourne, VIC 3000, Australia; 4 Department of Microbiology and Immunology, University of Melbourne, Parkville, VIC 3010, Australia

**Keywords:** tuberculosis, migration, child, epidemiology, elimination

## Abstract

Australia is a low tuberculosis incidence country. In the setting of increasing migration, we aimed to investigate the epidemiology and trends of tuberculosis in the Australian-born population in the state of Victoria between 1992 and 2017. We performed a retrospective descriptive analysis of demographic, clinical and outcome data extracted from a centralized notifiable disease database. The mean incidence of tuberculosis was 1.19 cases per 100,000 population per year with a small but significant reduction of 0.98% per year. The median age of cases decreased from 67.5 years in 1994 to 17 years in 2017. Among 0–14 year-olds, there was an increase from 0.13 cases per 100,000 population in 1996 to 2.15 per 100,000 population in 2017. Data for risk factors were available from 2002 onwards. The most common risk factor in the 0–14 year age group was a household contact with tuberculosis (85.1%), followed by having a parent from a high tuberculosis incidence country (70.2%). We found the rate of tuberculosis in the Australian-born population in Victoria is low. However, there has been an increase in incidence in children, particularly among those with links to countries with high tuberculosis incidence. This could threaten progress towards tuberculosis elimination in Australia.

## 1. Introduction

The World Health Organization post-2015 global tuberculosis (TB) strategy aims to reduce the global TB incidence by 90% to less than 10 cases per 100,000 population by 2035 [1]. For low incidence countries such as Australia, a framework has been set out to aim for elimination by 2050. This has been defined as an incidence of <1 case per million population [2].

Like other developed countries, Australia has had a dramatic reduction in TB incidence since the 1950s [3,4]. Despite investments in nationwide reporting, universal availability of treatment and diagnosis, contact tracing and migrant screening, the incidence of TB in Australia remains between 5 to 7 per 100,000 population per year (ppy) [3]. In fact, up to 2012, there had been an increase in incidence over the preceding decade [5].

Victoria is in the south-east of Australia. It is the second most populous state in Australia with a population of 6.36 million in September 2017 [6]. The incidence of TB in Victoria is comparable to the rest of Australia at 6.4 per 100,000 in 2013 [3]. In Victoria, current guidelines for contact tracing involve standardised interviews regarding extent of contact, with break-of-contact tuberculin skin testing or interferon-gamma release assays performed routinely three months following exposure. Additional testing is performed at baseline for household or other contacts at high risk. Case and contact management is conducted by the Victorian Tuberculosis Program, under the auspices of the Victorian Department of Health and Human Services.

Between 88% to 95% of TB cases in Australia have been reported in the overseas-born population [3,4]. Increasing migration from countries with high TB burden has been postulated to be responsible for the observed increase in incidence [3]. However, review of the TB epidemiology in the Australian-born population is also required as travel patterns may affect TB risk amongst this group.

In this study, we aimed to investigate the epidemiology of notified TB cases in Australian-born individuals in Victoria from 1992 to 2017, with a focus on demographic trends over time and on Australian-born children.

## 2. Materials and Methods

This retrospective descriptive study included cases born in Australia who were notified with active TB in Victoria between 1 January 1992 and 31 December 2017. In Australia, medical practitioners and pathology services are required by legislation to report cases of TB to public health authorities [3]. Notified cases in Victoria are followed up by the Victorian TB Program with case data collected and stored in a centralized database. Cases of active TB required confirmation by a positive culture or polymerase chain reaction, or had to meet clinical or radiological criteria as determined by a clinician experienced in the management of TB [7]. Countries were classified as high-incidence for TB if their annual incidence was greater than 40 cases per 100,000 population as estimated by WHO [8].

De-identified data were extracted from the Public Health Events Surveillance system, a centralized notifiable disease database. Demographics, ethnicity (self-reported), parental country of birth, manifestation, method of diagnosis, antimicrobial susceptibility, treatment outcome, and risk factors were collected. As the study used long-term surveillance data, only a subset of variables (age, gender, country of birth, region of residence, site of disease, and diagnosis method) were available for the entire study period. Other variables were available at varying time points due to data completeness and commencement of data collection. Metropolitan was defined as residing within the government-designated geographical catchment of Melbourne, the largest city in Victoria. Recurrence was defined as any case of TB occurring in a person with a previous history of treated TB (encompassing both relapse and reinfection). Persons lost to follow-up comprised individuals who should have completed treatment in Australia, but outcome was not known. Due to legislative restrictions, data on HIV–TB co-infection were only available from the TB database from 2009 onwards. TB-attributed death was reported using previously published definitions [9].

Incidence of active TB per 100,000 was calculated using Australian Bureau of Statistics census data. Population numbers stratified by country of birth and by age were only available on census years (1991, 1996, 2001, 2006, 2011, and 2016) [10,11,12,13,14,15]. The number of Australian-born persons but not age-specific data was available for census years 1991 and 1996. The proportion of people aged 0–14 years who were born in Australia compared to all ages was consistent on census years 2001, 2006, 2011, and 2016. The mean of these proportions was used to extrapolate 0–14 year Australian-born population data for census years 1991 and 1996. This was not applicable for other age groups and incidence was only calculated from 1999 for age groups other than 0–14 years. Incidence was calculated using census data from the closest year. For example, incidence in 1994 was calculated using data from the 1996 census. Poisson regression was used to assess trends in incidence rates. Incidence trends were analysed from 1992 to 2017 for the 0–14 year age group and from 1999 to 2017 for the 15–24, 25–34, 35–44, 45–54, 55–65, and over 65 year age groups. Data analysis was performed using Microsoft Excel 2016 (Microsoft, Redmond, WA, USA) and Stata version 13.0 (Stata Corp, College Station, TX, USA).

As data in this study were collected and used under the legislative authority of the Public Health and Wellbeing Act 2008, approval from a Human Research Ethics Committee was not required under the rules of our institutions.

## 3. Results

### 3.1. Demographics

There was a total of 1057 cases of TB reported during the study period (Table 1). Most cases were identified after clinical presentation, with 11.3% of cases (119/1057) found through active contact tracing. Data for Aboriginal or Torres Strait Islander status were not reliably recorded prior to 1998. From 1998 to 2017, there were 18 cases recorded in Aboriginal or Torres Strait Islander people (range 0–5 cases per year). 

Risk factors for TB acquisition were available from 2002 onwards. Data were available for 78.4% of cases during this period (485/619). The most common risk factor was a household contact with TB accounting for 49.1% of cases where data were available (238/485). This was followed by travel to a country with high TB incidence for at least three months (37.9%, 184/485) and having at least one parent from a country with high TB incidence (23.3%, 113/485). Parental origin was only measured as a risk factor in those aged 0–14 years. Homelessness was reported as a risk factor in 15 cases (3.1%, 15/485). No risk factor was reported in 4.9% of cases (24/485). 

Within the 0–14 year age group, 85.1% (137/161) had a household contact with TB, 70.2% (113/161) had a parent from a country with high TB incidence, and 19.3% (31/161) had travelled to a country with high TB incidence for at least three months. More than half (51.6%, 83/161) had at least one parent from a country with high TB incidence and a household contact but no high-risk overseas travel. All children had at least one of these risk factors. In the 0–14 year age group, 57.8% of cases were found by active contact tracing. This is in contrast to the over-50 year age group, where only 4.4% were found by active contact tracing.

Within the 15–24 year age group, risk factors were available for 89/100 cases. Of these, 77.5% (69/89) had a parent from a country with high TB incidence, 53.9% (48/89) had travelled to a country with a high TB incidence, and 37.1% (33/89) had a household contact. Only 4.5% (4/89) had no recorded risk factor.

Data for ethnicity or parental country of birth were only systematically recorded for all age groups from 2009 onwards. Data were available for 80% of cases during this period (272/340). From 2009 to 2017, 149 (54.8%) cases with active TB had an ethnic background or a parent from a high TB endemic country (Table 2). The region most commonly identified was Africa (60/149, 40.3%) followed by South East Asia (49/149, 32.9%). Based on data available for 60/91 (65.9%) cases in the over-50 year age group, only 1.7% (1/60) of cases in the over-50 year age group had an ethnic background or a parent from a high TB endemic country.

### 3.2. Clinical Characteristics

There were 1011 cases with manifestation of disease recorded (Table 1). Of the 94% of cases (991/1057) with method of diagnosis data available, over two-thirds of cases were culture confirmed (71.8%, 712/991). In cases 15 years and older, 80.1% of cases (650/804) were culture confirmed but it was only 33.2% (62/187) in the 0–14 year age group. The proportion of culture-confirmed cases did not change over the study period. Drug susceptibility results were available from 1998 onwards. The majority were fully drug susceptible to first-line drugs with resistance to one or more drugs reported in 36/555 cases (6.5%). There were eight cases of multidrug-resistant TB during this period. HIV testing results were available for 59.7% of cases from 2009 onwards (203/340). There were seven cases of HIV and TB coinfection.

### 3.3. Treatment Outcomes

Treatment outcome data were available from 2002 onwards (613 cases, Table 1). Excluding cases still on treatment and those who died of causes other than TB, 95% of cases had successfully completed treatment (508/528). Eight cases (1.3%) died from TB during treatment.

### 3.4. Trends

**Figure 1:**
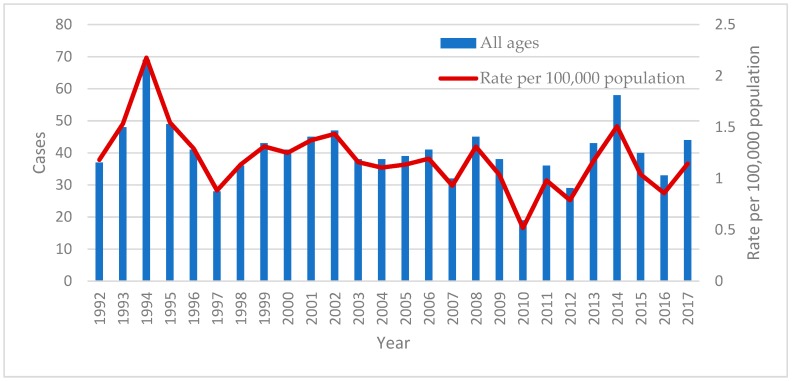
Number of cases and rate per 100,000 population of notified tuberculosis (TB) cases between 1992 and 2017.

The mean incidence of active TB over the period of the study was 1.19 cases per 100,000 ppy. The peak incidence was in 1994 with 2.18 cases per 100,000 and the lowest was 0.52 cases per 100,000 in 2010. Overall there a small but significant reduction in the incidence over the study period of 0.98% per year (*p* < 0.0001, Figure 1).

**Figure 2:**
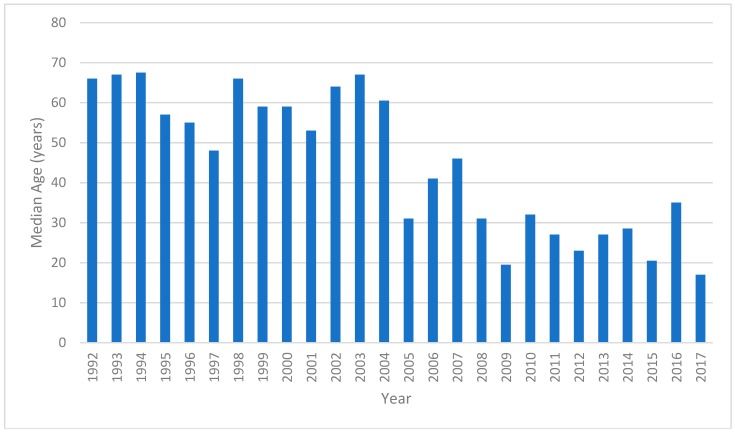
Median age of notified TB cases between 1992 and 2017.

**Figure 3:**
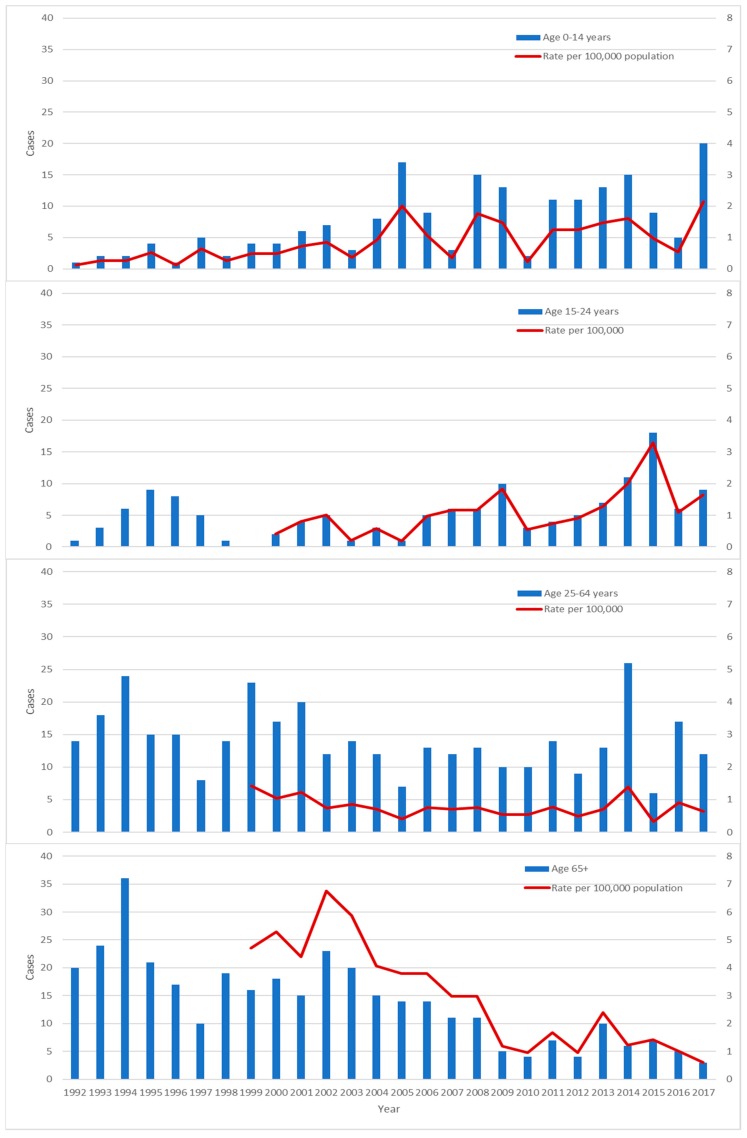
Number of cases per year and incidence per year by age group.

The median age of cases peaked at 67.5 years in 1994 and had declined to 17 years in 2017 (Figure 2). There was a total of 192 cases in 0–14 year-olds during the period of the study. The mean incidence in 0–14 year-olds was 0.85 cases per 100,000 ppy. There was an increase in incidence through the study period (*p* < 0.0001, Figure 3). The lowest incidence was in 1996 with 0.13 cases per 100,000 ppy. The peak was recorded in 2017 with 2.15 cases per 100,000 ppy. There was a similar increase in the 15–24 year age group (*p* < 0.0001) peaking at 3.28 cases per 100,000 population in 2015. Concordant with the decline in median age of cases, there was a decline in incidence in the >65 year age group from 6.75 cases per 100,000 ppy in 2002 to 0.61 cases per 100,000 ppy in 2017 (*p* <0.0001). There was no significant change in incidence in other age groups.

## 4. Discussion

This study demonstrates low rates of tuberculosis among the Australian-born population in Victoria but an increase in incidence in the younger age groups, particularly in children. The mean incidence between 1992 and 2017 was 1.19 cases per 100,000 which is just above the pre-elimination target of <1 per 100,000 ppy [2].

An increasing incidence among the 0–14 year and 15–24 year subgroups could threaten the progress towards the elimination target of <1 case per million ppy. Incidence of TB in children is an indicator of recent transmission and is an important performance indicator for progress towards elimination [3]. There was an increase in incidence in the 0–14 year age group from 0.13 cases per 100,000 in 1996 to 2.15 cases per 100,000 in 2017. Taken collectively with an increase in the 15–24 year age group, this suggests there has also been an increase in incidence amongst adolescents who may pose a higher risk of transmission than children, who tend to have pauci-bacillary disease.

This increase has been coincident with a reduction in the median age of cases from 67.5 years in 1994 to 17 years in 2017. Within the Australian-born population, there is a peak in incidence of TB in the older (>65-year) age group [3,4,16]. This reflects low rates of TB transmission in Australia in recent years [17,18,19] and reactivation of latent TB in the older population. The decline in incidence of active TB in the older Australian-born population has been noted previously [9]. 

Within the 0–14 year subgroup, the majority had a household contact with active TB and more than half of the cases were found by contact tracing. These were slightly higher than reported in a study in children in Australia from 2003 to 2012 [20]. This rate is also much higher than the 4.4% found in the over-50 year age group and that reported previously [4]. In addition, more than two thirds of cases within the 0–14 year age group had an ethnic background or parent from a high TB incidence country and almost 20% had spent three or more months living in a high TB incidence country. It has previously been identified that most cases of active TB in Australia are among the overseas-born [3,4,16,21]. These results suggest that acquisition in the 0–14 year subgroup may be related to limited transmission of TB within families where adults born overseas develop reactivated TB and to lesser extent, acquisition from travel to high TB incidence countries.

The risk factors identified in the 15–24 year age group were similar with the majority having a parent from a high TB incidence country. However, within this group, there was a higher rate of travel to high TB incidence countries (53.9%) and less than half had a household contact (37.1%). This suggests that travel may be a more important risk factor in this age group. The most commonly identified regions of ethnicity or parental birthplace were Africa and Southeast Asia, which combined to constitute over 70% of cases where data were available. This mirrors previously-reported data in Australia-born children with TB [20]. Interestingly, South Asia constituted only just over 10% of cases. In adults born overseas, South Asia has been the most commonly identified region of origin [3]. The reason for this difference between overseas-born adults and Australian-born children is not clear from this data.

The increase in incidence among the Australian-born children was not identified in an Australia-wide epidemiological report up to the year 2013 [3]. Ethnicity or parental country of birth in the Australian-born population was not reported in the Australia-wide report and risk factors were not reported by age. In 2015–2016, Victoria recorded the highest net migration of any state in Australia and the second highest net overseas migration of over 65,000 people [22]. Within Victoria, the reported number of people with both parents born overseas was higher than the Australian average in both 2016 (38.5% vs. 34.4%) [15] and 2011 (38.1% vs. 34.3%) [14]. Therefore, findings in Victoria, in the setting of high migration, may demonstrate a sentinel trend before it becomes evident nationwide.

The shifting epidemiological trend in active TB cases in the Australian-born from older Australians to younger persons with links to high TB incidence countries has implications for TB control in Australia. This highlights the need for maintaining efforts in TB reporting and contact tracing and increasing provider awareness to reduce diagnostic delays. Ongoing surveillance should continue to capture data on second generation migrants including information on parental country of birth and risk factors.

This also suggests that there may be an opportunity to prevent transmission to the higher risk 0–14 year and 15–24 year subgroups by preventing TB reactivation within their family. Screening and treatment of latent tuberculosis has been considered as a strategy to reduce the incidence of TB reactivation in migrants to Australia [23,24]. Current Australian refugee health guidelines suggest screening for latent tuberculosis in people aged ≤35 years [25]. The findings of this study add weight to this strategy to reduce transmission to second-generation, Australian-born, children.

The BCG vaccine is currently recommended in Australia for children travelling to high TB incidence countries for extended periods [26]. Prior to the mid-1980s, BCG vaccination was offered to all school children except in two states (New South Wales and the Australian Capital Territory) [26]. While BCG vaccination status was not captured in the study, prioritization of the risk groups identified in this study for assessment of BCG vaccination could also be considered to reduce TB disease incidence and/or severity.

The strength of this study is the capture of a comprehensive population-based data set from a notifiable centralized database over a long observation period. However, data from all fields were not complete, limiting analysis in some categories to only a subset of years. Our analysis is also limited by its retrospective nature.

## 5. Conclusions

The rates of TB in the Australian-born population in Victoria are low. However, there is an epidemiological shift with an increasing rate of TB in children with parents from countries of high TB incidence and decreasing incidence in the older Australian-born population. This could threaten progress towards elimination of TB in Australia. This reinforces the importance of TB control efforts with reporting and contact tracing in Australia and consideration of screening of high risk groups for latent TB to prevent reactivation and local transmission to vulnerable subgroups such as children.

## Figures and Tables

**Table 1:** Selected characteristics of tuberculosis (TB) cases in the Australian-born in Victoria between 1992 and 2017.

Characteristic	All Ages	0–14 Years	≥15 Years
**Number of cases**	1057	192	865
**Median age (IQR)**	47 years (20, 73)	3 years (2, 7)	57 years (31, 76)
**Male (%)**	618 (58.5)	106 (55.2)	512 (59.2)
**Metropolitan (%)**	830 (78.5)	178 (92.7)	652 (75.4)
**HIV co-infection ^1^**	7	0	7
**Method of diagnosis (%)**			
Culture	712 (67.4)	62 (32.3)	650 (75.1)
Radiologic	145 (13.7)	96 (50.0)	49 (5.7)
Nucleic acid testing/microscopy	61 (5.8)	14 (7.3)	47 (5.4)
Histology	39 (3.7)	3 (1.6)	36 (4.2)
Clinical	34 (3.2)	12 (6.3)	22 (2.5)
Missing	66 (6.2)	5 (2.6)	61 (7.1)
**Multi-drug resistance ^2^**	8	1	7
**Manifestation (%) ^3^**			
Pulmonary only	610 (57.7)	106 (55.2)	504 (58.3)
Pulmonary and extrapulmonary	128 (12.1)	25 (13.0)	103 (11.9)
Miliary	20 (1.9)	6 (3.1)	14
Extrapulmonary only	273 (25.8)	59 (30.7)	214 (24.7)
Extrapulmonary manifestations			
Lymphadenitis	127 (12.0)	50 (26.0)	77 (8.9)
Pleural	105 (9.9)	6 (3.1)	99 (11.4)
Bone/joint	38 (3.6)	5 (2.6)	33 (3.8)
Genitourinary	33 (3.1)	1 (0.5)	32 (3.7)
Meningeal	18 (1.7)	9 (4.7)	9 (1.0)
Cutaneous	7 (0.7)	1 (0.5)	6 (0.7)
Pericardial	5 (0.5)	0	5 (0.6)
Other	5 (0.5)	2 (1.0)	3 (0.3)
**Recurrence (%)**	13 (1.2)	2 (1.0)	11 (1.3)
**Treatment outcomes ^4^**			
Median duration (IQR)	212 days (184, 278)		
Completed treatment	508	140	368
Still on treatment	36	18	18
Death during treatment	57	0	57
Death from TB	8	0	8
Default	4	1	3
Lost to follow-up	8	2	6

^1^ Data available in 203/340 cases from 2009 onwards; ^2^ Data from 555 cases with drug susceptibility results from 1998 onwards; ^3^ Manifestation data missing in 46 cases (2 in 0–14 year group, 44 in ≥15 year group); ^4^ Data from 2002 onwards—613 cases during this period (161 in 0–14 year group, 452 in ≥15 year group); IQR = interquartile range.

**Table 2:** Ethnic identification or country of birth of parent from 2009 to 2017.

Country or Region	Number (%)
Total	149
Africa	60 (40.3)
Sudan	24 (16.1)
Somalia	25 (16.8)
Ethiopia	9 (6.0)
Algeria	1 (0.7)
Zimbabwe	1 (0.7)
Southeast Asia	49 (32.9)
Vietnam	26 (17.4)
Philippines	9 (6.0)
Indonesia	3 (2.0)
Malaysia	2 (1.3)
Timor-Leste	2 (1.3)
Laos	3 (2.0)
Cambodia	3 (2.0)
Myanmar	1 (0.7)
North Asia	7 (4.7)
China	6 (4.0)
Tibet	1 (0.7)
South Asia	15 (10.0)
India	14 (9.4)
Pakistan	1 (0.7)
Middle East	8 (5.4)
Afghanistan	7 (4.7)
Iraq	1 (0.7)
Pacific Islands	9 (6.0)
Tonga	4 (2.7)
Samoa	4 (2.7)
Fiji	1 (0.7)
South America	
Peru	1 (0.7)
